# *MSR1* is not required for obesity-associated inflammation and insulin resistance in mice

**DOI:** 10.1038/s41598-023-29736-0

**Published:** 2023-02-14

**Authors:** Sierra A. Nance, Lindsey Muir, Jennifer Delproprosto, Carey N. Lumeng

**Affiliations:** 1 Molecular and Integrative Physiology, University of Michigan Medical School, 109 Zina Pitcher Place, 2057 BSRB, Ann Arbor, MI 48109 USA; 2 Department of Pediatrics, University of Michigan Medical School, 109 Zina Pitcher Place, 2057 BSRB, Ann Arbor, MI 48109 USA; 3 Computational Medicine and Bioinformatics, University of Michigan Medical School, Ann Arbor, MI USA

**Keywords:** Inflammation, Metabolism

## Abstract

Obesity induces a chronic inflammatory state associated with changes in adipose tissue macrophages (ATMs). Macrophage scavenger receptor 1 (*MSR1*) has been implicated in the regulation of adipose tissue inflammation and diabetes pathogenesis; however, reports have been mixed on the contribution of MSR1 in obesity and glucose intolerance. We observed increased *MSR1* expression in VAT of obese diabetic individuals compared to non-diabetic and single nuclear RNA sequencing identified macrophage-specific expression of *MSR1* in human adipose tissue. We examined male *Msr1*^*−/−*^ (*Msr1*KO) and WT controls and observed protection from obesity and AT inflammation in non-littermate *Msr1*KO mice. We then evaluated obese littermate *Msr1*^*+/−*^ (*Msr1*HET) and *Msr1*KO mice. Both *Msr1KO* mice and *Msr1HET* mice became obese and insulin resistant when compared to their normal chow diet counterparts, but there was no *Msr1*-dependent difference in body weight, glucose metabolism, or insulin resistance. Flow cytometry revealed no significant differences between genotypes in ATM subtypes or proliferation in male and female mice. We observed increased frequency of proliferating ATMs in obese female compared to male mice. Overall, we conclude that while *MSR1* is a biomarker of diabetes status in human adipose tissue, in mice *Msr1* is not required for obesity-associated insulin resistance or ATM accumulation.

## Introduction

Type 2 Diabetes (T2D) is a metabolic disorder characterized by insulin resistance and hyperglycemia and is a growing health concern. Obesity is the number one modifiable risk factor for T2D^1,2^. The association between obesity and T2D is well established, but the mechanisms that link the two remain incompletely understood. Adipose tissue is a key player in regulating insulin sensitivity and nutrient storage^3^. It is composed of adipocytes and non-adipocytes, including leukocytes such as adipose tissue macrophages (ATM)^4^. Under lean conditions, there is a balance between adipokines and anti-inflammatory cytokines to maintain metabolic homeostasis. Obesity induces adipose tissue dysfunction characterized by a state of chronic inflammation and adipocyte insulin resistance^5–7^. ATMs are a primary mediator of cytokine production in adipose tissue, producing pro-inflammatory cytokines that have been shown to inhibit glycogen synthesis^8^, decrease glucose uptake^9^ and impair insulin signaling^10,11^ impacting systemic insulin resistance. These findings along with animal models showing that impaired macrophage activation improved metabolism with obesity suggests that ATMs have a direct effect on obesity-induced insulin resistance.

However, many gaps remain in understanding the beneficial and detrimental effects of ATM functions and how these functions are regulated. Along with a quantitative increase in the number of ATMs with obesity and insulin resistance, the expression of macrophage scavenger receptor (*MSR1*/Scavenger receptor A) also increases in adipose tissue^12^ suggesting that *MSR1* plays a role in promoting T2D. *MSR1* expression in subcutaneous adipose tissue (SAT) is associated with insulin resistance in non-diabetic patients^13^**.** GWAS studies indicate associations between SNPs in *MSR1* with waist-to-hip ratio and hip circumference adjusted for BMI^14^. The mechanism for this association is unknown.

*MSR1* is a scavenger receptor prevalent in macrophages that binds many ligands, including low-density lipoproteins^15^. *MSR1* is thought to aid in phagocytosis^16,17^ and promote inflammation in macrophages^18,19^. *Msr1* has been shown to play a role in macrophage proliferation in peritoneal macrophages from *Ldlr*^*/−*^ mice and in macrophages in atherosclerotic lesions in *Apoe*^*−/−*^ mice^20,21^. Similarly, in adipose tissue, both M2-like resident ATMs (CD11c^−^) present in lean states and recruited M1-like proinflammatory ATMs (CD11c^+^) proliferate robustly with acute and chronic high-fat diet-induced obesity^22,23^. The persistent proliferation of CD11c^+^ ATMs contributes to the long-term effects of obesity on adipose tissue function even after weight loss^24^. The factors that regulate ATM proliferative signals are not known.

How or if *MSR1* may play a role in ATM function is not well understood. Despite the clinical association between *MSR1* expression and T2D, there is evidence from mouse studies suggesting that *Msr1* may regulate beneficial macrophage functions to protect against obesity-induced insulin resistance. *Msr1*^−/−^ *ob/ob* mice were found by Zhu et al. to be more insulin resistant and glucose intolerant compared to *Msr1*^+/+^ *ob/ob* mice^25^. Similar worse insulin resistance was observed in *Msr1*^−/−^ mice fed HFD for 16 weeks. Cavallari et al.showed impaired glucose tolerance in obese (6-week HFD) *Msr1*^−/−^ mice compared to *Msr1*^+/+^ mice^26^. On the other hand, Govaere et al.reported that obese *Msr1*^*−/−*^ mice have increased weight gain, but are protected from obesity-induced insulin resistance, adipose tissue inflammation, and hepatic steatosis suggesting a pro-inflammatory role for *Msr1*^27^.

Because obesity is associated with macrophage accumulation and chronic inflammation, Zhu et al. assessed ATMs to explain their metabolic phenotype in HFD *Msr1*^+/+^ and *Msr1*^−/−^ mice and showed a decrease in resident CD11c^−^ ATMs and an increase in proinflammatory CD11c^+^ ATMs in *Msr1*^−/−^ *ob/ob* mice^25^. However, Callivari et al.did not note any significant differences in adipose tissue inflammation in their model and observed that administration of *MSR1* agonists worsened glucose tolerance. Conversely, Zhu et al. suggested protective effects of Msr1 activation^25,26^. Govaere et al. showed that Msr1 promotes lipid accumulation and hepatic macrophage activation, suggesting a role for *Msr1* in promoting obesity-induced inflammation. Furthermore, inhibition of *Msr1* reduced fibrosis and macrophage activation in mouse and human liver, identifying *Msr1* as a potential target for treating obesity-associated inflammation and liver damage^27^. Together, these results suggest that *MSR1* may play a role in obesity-associated insulin resistance and adipose tissue inflammation, but the precise mechanisms are unclear.

We noted that weakness of all of these studies was that littermate controls were not utilized for comparisons and female mice were not assessed. Using littermate controls has been shown to be a more rigorous approach for analysis of genetically modified mice as the use of non-littermate parallel colonies can have epigenetic and environmental backgrounds that can skew results^28^. In diabetes research, the lack of littermate controls has been suggested to be a significant confounding variable in interpreting metabolic phenotypes in mice^29^.

To address these gaps and evaluate the *hypothesis* that *MSR1* promotes ATM proliferation, we set out to examine the role for *Msr1* in diet induced obesity in mice and humans. We observed associations with *MSR1* expression in visceral adipose tissue (VAT) in obese diabetic patients and confirmed that *MSR1* is highly restricted to ATMs in human adipose tissue in single nuclear RNAseq analysis. Initial studies suggested protection from diet induced obesity in *Msr1*^*−/−*^ mice compared to non-littermate controls. However, comparison of littermate male and female mice did not demonstrate significant differences in obesity, glucose intolerance, or adipose tissue inflammation between genotypes. Our studies suggest that *MSR1* is a strong ATM biomarker in human adipose tissue associated with T2D status, but our data does not support a functional role for *Msr1* in regulating metabolic inflammation in mice.

## Methods & materials

### Animals and diet

*Msr1*^+*/−*^ (*Msr1*HET) mice were mated with *Msr1*^*−/−*^* (Msr1KO)* mice (Jackson Laboratory; Strain 006,096) to produce experimental *Msr1*^*−/−*^ pups and *Msr1*^+*/−*^ littermate controls. Pups were weaned at 21 days and assessed for sex. Mice were separated into four experimental groups: 1) *Msr1*^+*/−*^ on normal chow diet (ND) (10% fat; Research Diets: D12450J), 2) *Msr1*^*−/−*^ mice on ND, 3) *Ms1r*^+*/−*^ on a high-fat diet (HFD) (60% fat; Research Diets: D12492), 4) *Msr1*^*−/−*^ on HFD. Each group was fed a ND until 8 weeks of age at which time groups 3 and 4 were switched to HFD. All groups had free access to food and water. Weight was obtained at the final endpoint. For each time point, each group of mice was euthanized, and adipose tissue was collected for analysis. For non-littermate control experiments, *Msr1*^*−/−*^* (Msr1KO)* mice (Jackson Laboratory; Strain 006,096) were compared with age-matched C57BL/6 J male mice. For non-littermate experiments, Msr1KO and WT mice were housed in separate cages. For littermate experiments, Msr1HET and Msr1KO littermate mice were housed in the same cages. All animal protocols were approved by the University of Michigan Institutional Animal Care and Use Committee. All methods were performed in compliance with ARRIVE guidelines, the NIH Public Health Service Policy on Human Care and Use of Laboratory Animals, and the NIH Guide for the Care and Use for Animals.

### Glucose & insulin tolerance testing

Mice were fasted for 6 h and injected intraperitoneally (i.p.) with 1.0 g/kg of 10% glucose for the glucose tolerance test (GTT) or 1.0U/kg insulin for insulin tolerance test (ITT). Glucose levels were assessed at baseline and then every 15 min for 2 h using the *Freestyle Freedom Lite* glucometer and test strips.

### Flow cytometry

Mice were euthanized and adipose tissue depots and liver were dissected at the end of the experiment. Adipose tissue was subjected to collagenase digestion to isolate the stromal vascular fraction (SVF) as described^30^. SVF was incubated with antibodies to identify macrophage subsets and proliferation and analyzed by flow cytometry on the BD Biosciences LSRFortessa™ cell analyzer as described^30^. The following conjugated antibodies were used: CD301 APC (Clone ER-MP23, BioRad, Cat# MCA2392A647), CD45 Pacific Blue (Clone 30-F11, eBiosciences, Cat# 48,045,182), CD64 PE (Clone X54-6/7.1.1, BD Biosciences, Cat# 558,455), CD11c APC-Cy7 (Clone N418, eBiosciences, Cat# 47,011,482), and Ki67 PeCy7 (Clone SolA15, eBiosciences, Cat#25,569,882). Single-stained, isotype, and unstained SVF were used as controls.

### Gene expression

Total RNA was obtained from frozen mouse white adipose tissue via mechanical homogenization using the TissueMiser homogenizer (Fisher Scientific) and isolated using Qiagen’s Rneasy® Mini Kit (Cat# 74,106; Qiagen, Hilden, Germany). RNA was treated with DNase I (Cat# 79,254; Qiagen, Hilden, Germany)) and cDNA was prepared using 9.8-20 ng total RNA and Applied Biosciences™ High-Capacity cDNA Reverse Transcription Kit (Cat# 4,368,814; Thermo Fisher Scientific, Waltham, MA, USA). Transcript expression was measured using SYBR Power Green PCR Master Mix (Cat# 4,367,659; Thermo Fisher Scientific, Waltham, MA, USA) in a Quantstudio™ 3 real-time PCR cycler (Applied Biosystems by Thermo Fisher Scientific, Waltham, MA, USA). Target genes were compared to the geometric mean of the housekeeping gene, *Arbp* using the ΔΔ*C*_*T*_ method. Gene expression was analyzed in *Msr1*KO (n = 5) and *Msr1*HET mice (n = 3). Supplementary Table 1 lists primers for qPCR.

### RNA sequencing analysis

The Genotype-Tissue Expression (GTEx) Project was supported by the Common Fund of the Office of the Director of the National Institutes of Health, and by NCI, NHGRI, NHLBI, NIDA, NIMH, and NINDS. Analysis of MSR1 expression was performed using negative binomial regression model including sex, age and BMI in the model in R. The data used for the GTEX described in this manuscript are available at dbGaP accession number phs000424.vN.pN on 11/12/2022.

## Results

### MSR1 is macrophage-specific and is increased in visceral adipose tissue from obese diabetic humans

**Figure 1:**
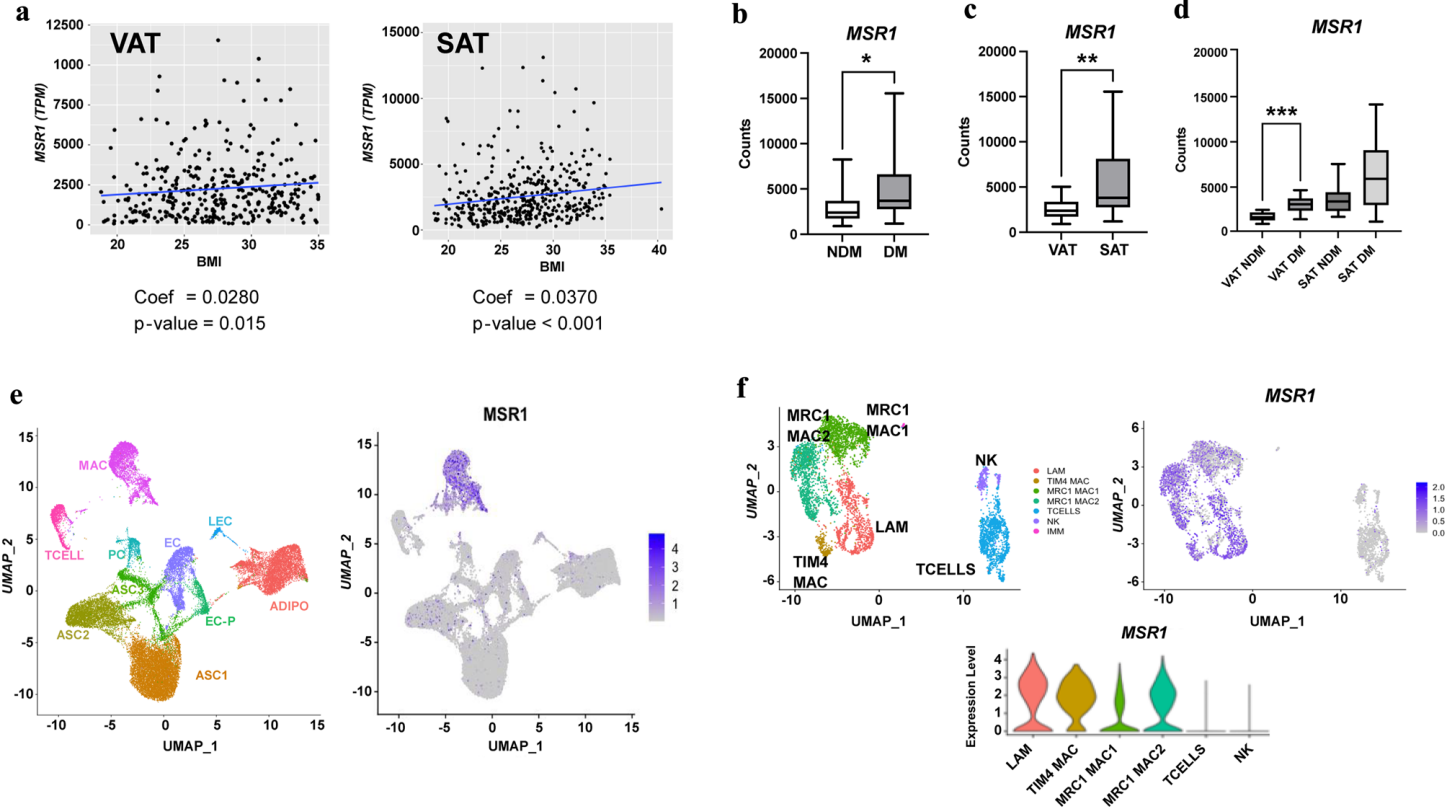
MSR1 gene expression is increased in diabetic visceral adipose tissue in humans. (**a**) Correlation between *MSR1* expression and BMI in omental/visceral (VAT; n = 354) and subcutaneous (SAT; n = 439) adipose tissue in GTEX. Negative binomial model used incorporating BMI, sex, and age in the model. (**b**–**d**) *MSR1* expression from RNAseq data from independent cohort of VAT and SAT samples (n = 40). Corrected counts reported comparing (**b**) non-diabetic (NDM) and diabetic (DM), (**c**) VAT and SAT, and (**d**) depots stratified by DM status. *p-value < 0.05. ** *p*-value < 0.01. ***p-value < 0.001. (**e**) UMAP plots from single nuclear RNAseq data from human VAT and SAT identifying major cell types. *MSR1* expression shown restricted to macrophages (MAC) cells. (**f**) Subcluster analysis of macrophages and T cells from single nuclear RNAseq analysis identifying lipid associated macrophages (LAM), TIM4 macrophages, and two types of MRC1 macrophages. MSR1 expression identified in all ATM subtypes.

To assess the expression of *MSR1* in human adipose tissue, we first interrogated the Genotype-Tissue Expression (GTEx) Project database for MSR1 expression in bulk RNA sequencing data. Adjusting for sex and age, *MSR1* expression in both omentum/visceral adipose tissue (VAT) and subcutaneous adipose tissue (SAT) were significantly associated with BMI (VAT: Coefficient = 0.0280, *p*-value = 0.015; SAT Coefficient = 0.0370, *p*-value < 0.001) (Fig. 1a). We have previously published analysis of bulk RNA sequencing data from VAT and SAT from obese patient with and without diabetes mellitus (DM). Comparing non-DM (NDM) with DM patients, *MSR1* expression was significantly increased in adipose tissue of DM patients. (Fig. 1b) Further stratifying the dataset by depot identified significantly higher *MSR1* expression in SAT compared to VAT (Fig. 1c). Stratifying by depot and DM status, we observed higher expression of *MSR1* in DM patients in VAT, but not SAT suggesting that MSR1 is a VAT specific biomarker of DM in humans (Fig. 1d).

To identify the cell types that express MSR1 in human adipose tissue, we interrogated a single nuclear RNAseq dataset we generated from obese human adipose tissue^31^. *MSR1* expression was exclusive to adipose tissue macrophages (MAC) and not seen in other stromal cell types (Fig. 1e). Similar results were observed in interrogation of murine and human single nuclear RNA sequencing studies by Emont et al.^32^*.* Subset analysis of ATMs identified multiple subtypes including lipid-associated macrophages (LAM). MSR1 was expressed in all ATM subtypes and was not significantly enriched in one ATM type compared to others (Fig. 1f). This data indicates that *MSR1* is a biomarker of all ATMs in human adipose tissue.

### Msr1KO mice are protected from HFD-induced obesity compared to non-littermate controls

**Figure 2:**
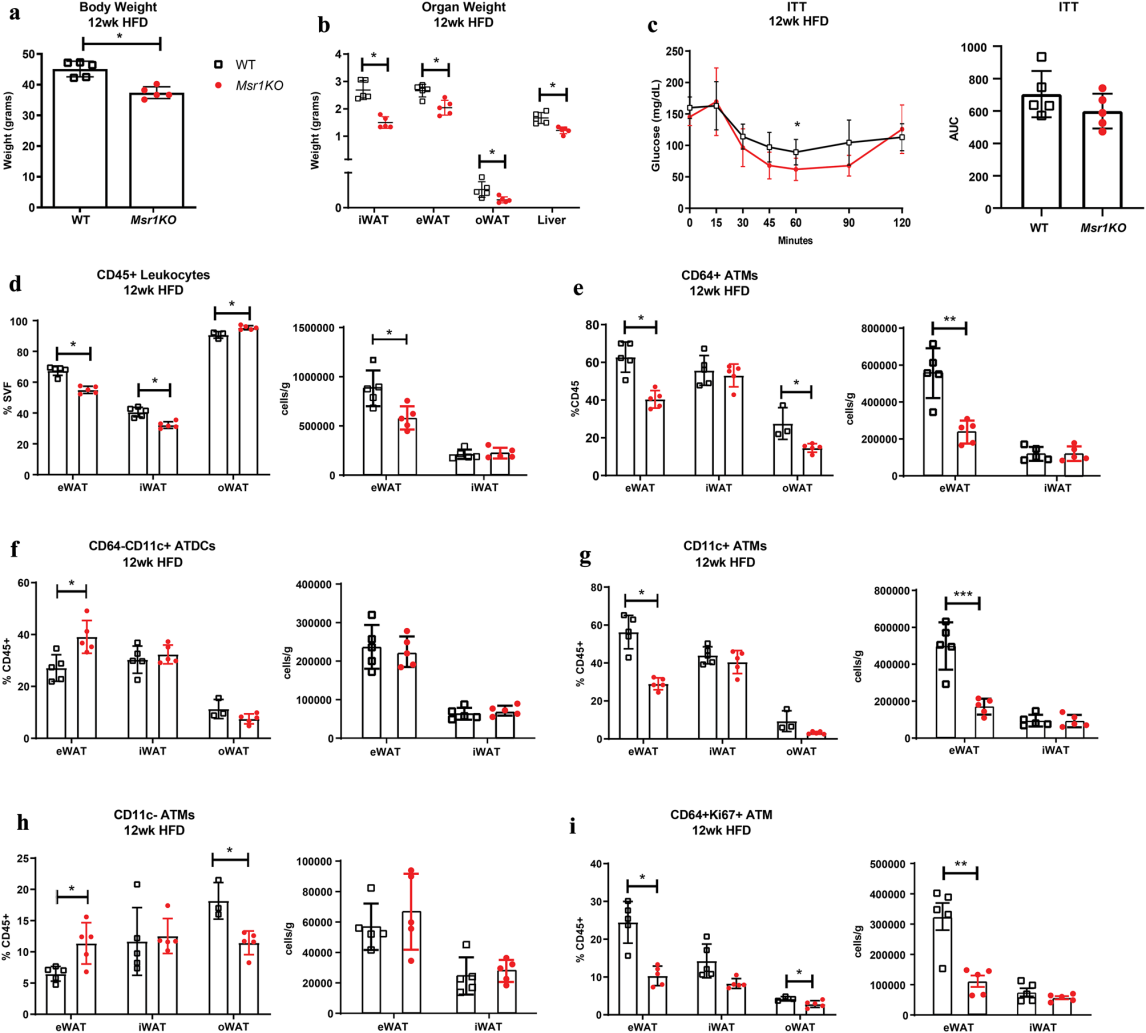
Male Msr1KO mice have decreased fat mass and inflammation compared to non-littermate wild-type controls. (**a**) Whole body weight for 12-week HFD fed *Msr1KO* (red) and C57BL6 wild type non-littermate controls (white). (**b**) Organ weight for *Msr1KO* (red) and WT non-littermate controls (black). (**c**) Insulin tolerance test (ITT) after 12 weeks of HFD. Flow cytometry analysis of (**d**) CD45^+^ leukocytes, (**e**) CD64^+^ ATMs, (**f**) CD64^−^CD11c^+^ ATDCs, (**g**) CD11c^+^ ATMs, (**h**) CD11c^+^ ATMs, and (**i**) CD64^+^Ki67^+^ ATMs in SVF after 12 weeks of HFD. n = 5/group. *p < 0.05. Statistical Analysis = T-test. Identical results observed when data was expressed as percent total SVF cells.

We performed experiments to compare the impact of high fat diet induced obesity on age matched WT and *Msr1*KO male mice maintained as parallel lines. After 12 weeks of HFD, *Msr1*KO mice had significantly less body weight and smaller fat pads compared to WT controls (Fig. 2a, b). While fasting glucose levels were similar, insulin tolerance tests demonstrated no significant differences in insulin sensitivity in *Msr1*KO male mice compared to controls (Fig. 2c). To assess whether *Msr1* was required for obesity-induced adipose tissue inflammation, flow cytometry was performed on the SVF from three fat pads: epididymal white adipose tissue (eWAT), inguinal white adipose tissue (iWAT), and omental white adipose tissue (oWAT). *Msr1*KO mice had a lower frequency of CD45^+^ leukocytes in eWAT and iWAT, but more leukocytes were seen in *Msr1*KO oWAT (Fig. 2d). *Msr1KO* mice also had fewer total CD64^+^ ATMs in eWAT and oWAT, but not iWAT (Fig. 2e). When we assessed adipose tissue dendritic cells (ATDCs) and ATM subtypes we found that *Msr1KO* mice had an increase in CD64^−^CD11c^+^ ATDCs and resident CD64^+^CD11c^−^ ATMs in eWAT compared to WT controls while inflammatory CD64^+^CD11c^+^ ATMs decreased (Fig. 2f, h). The frequency of resident CD64^+^CD11c^−^ ATMs was also lower in oWAT, indicative of depot-specific differences. We observed fewer CD45^+^CD64^+^Ki67^+^ proliferating ATMs in eWAT and oWAT, but not iWAT from *Msr1*KO mice after 12 weeks of HFD (Fig. 2i). These results suggest that *Msr1* is necessary for weight gain with HFD induced obesity and may be required to sustain obesity-associated inflammation. Our data also suggest a role for *Msr1* in regulating adipose tissue inflammation via promoting ATM proliferation.

### Msr1KO mice are not protected from HFD-induced obesity compared to Msr1HET littermate controls

**Figure 3:**
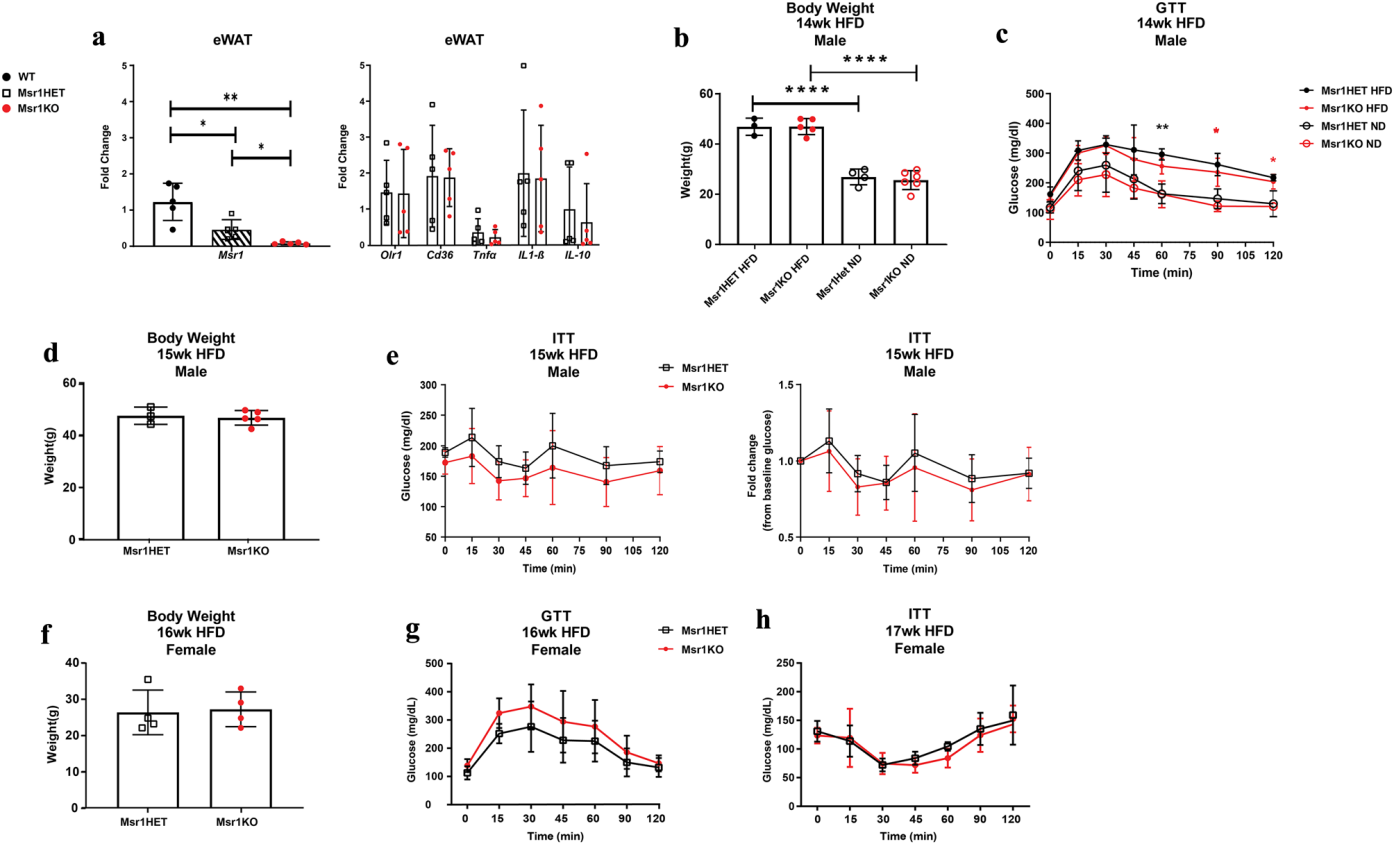
Msr1 is not required for obesity-induced changes in glucose metabolism (**a**) Gene expression of Msr1, scavenger receptors, and cytokines in iWAT and eWAT by qPCR from male *Msr1HET* (black) and *Msr1KO* (red) male mice. n = 5/group. *p < 0.05; Statistical Analysis = t-test. WT mice were non-littermate HFD fed controls. (**b**) Whole body weight for 14-week male ND and HFD-fed *Msr1HET* and *Msr1KO* mice at the time of (**c**) glucose tolerance test (GTT). (**d**) Whole body weight for 15-week male HFD-fed mice at the time of (**e**) Insulin tolerance test (ITT). n = 3–5 per group. (**f**) Whole body weight for 16-week female HFD-fed *Msr1HET* (black) and *Msr1KO* (red) female mice. (**g**) Glucose tolerance test (GTT) and (**h**) insulin tolerance test (ITT) after 16 and 17 weeks of HFD, respectively. HFD *Msr1HET* (black), HFD *Msr1KO* (red). n = 4/group. Statistical Analysis for weight = Ordinary one-way ANOVA. ****p < 0.0001 between *Msr1KO* ND and HFD groups; Statistical Analysis for GTT/ITT = 2-way ANOVA multiple comparisons. **p < 0.05 between ND and HFD groups of the same genotype.

As these results contrasted with prior reports of worse insulin resistance in *Msr1*KO mice, to improve the rigor of the study, we compared *Msr1*KO mice and littermate *Msr1*HET controls. COVID-19 pandemic restrictions did not allow us to examine littermate *Msr1HET*, *Msr1KO*, and *Msr1* + */* + littermates and the lines were subsequently lost. To justify the use of *Msr1*HET mice as a control, qRT-PCR was performed from eWAT samples demonstrating loss of *Msr1* expression in the KO mice and ~ 2 lower expression in HET mice compared to WT controls (Fig. 3a). Adipose tissue was harvested from experimental *Msr1*KO and *Msr1*HET littermate control male mice and analyzed for *Msr1* expression, along with scavenger receptors *Olr1* and *Cd36*, and cytokines *Tnfα, IL-10,* and *IL1β* by qPCR. *Msr1KO* mice had significantly less expression of *Msr1* in eWAT than *Msr1HET* mice *(*Fig. 3a*).* Gene expression of *Olr1, Cd36, Tnfα, IL-10,* and *IL1β* was not different between the two groups. This data demonstrated that *Msr1*KO mice and *Msr1*HET mice are differentiated by *Msr1* expression, making *Msr1*HET an appropriate control to explore the requirement of *Msr1* in obesity-associated inflammation and insulin resistance.

To evaluate the role of *Msr1* on obesity-associated impaired glucose metabolism and insulin resistance, 8-week-old male *Msr1KO* and *Msr1HET* mice were fed HFD to induce obesity, then assessed by glucose (GTT) and insulin tolerance tests (ITT) at 14 and 15 weeks HFD, respectively. Both HFD groups gained significantly more weight than ND mice, but there were no differences in body weight between genotypes for ND or HFD-fed mice. (Fig. 3b). GTT showed that HFD-fed mice had worse glucose tolerance compared to ND fed mice but there were no differences between genotypes (Fig. 3c). HFD groups of mice were assessed for body weight and ITT at 15 weeks of HFD. Again, there was no difference in body weight or ITT responses between HFD *Msr1*HET and *Msr1*KO mice (Fig. 3d, e).

We also assessed response to HFD in female *Msr1*HET and *Msr1*KO mice. Both genotypes had similar weights after 16 weeks of HFD (Fig. 3f). Similar to male mice, there were no significant differences in GTT or ITT between genotypes after HFD induced obesity (Fig. 3g, h). This data suggests that *Msr1* is not required for weight gain and insulin resistance during diet-induced obesity in either male or female mice.

### Msr1KO is not required for ATM accumulation with HFD

**Figure 4:**
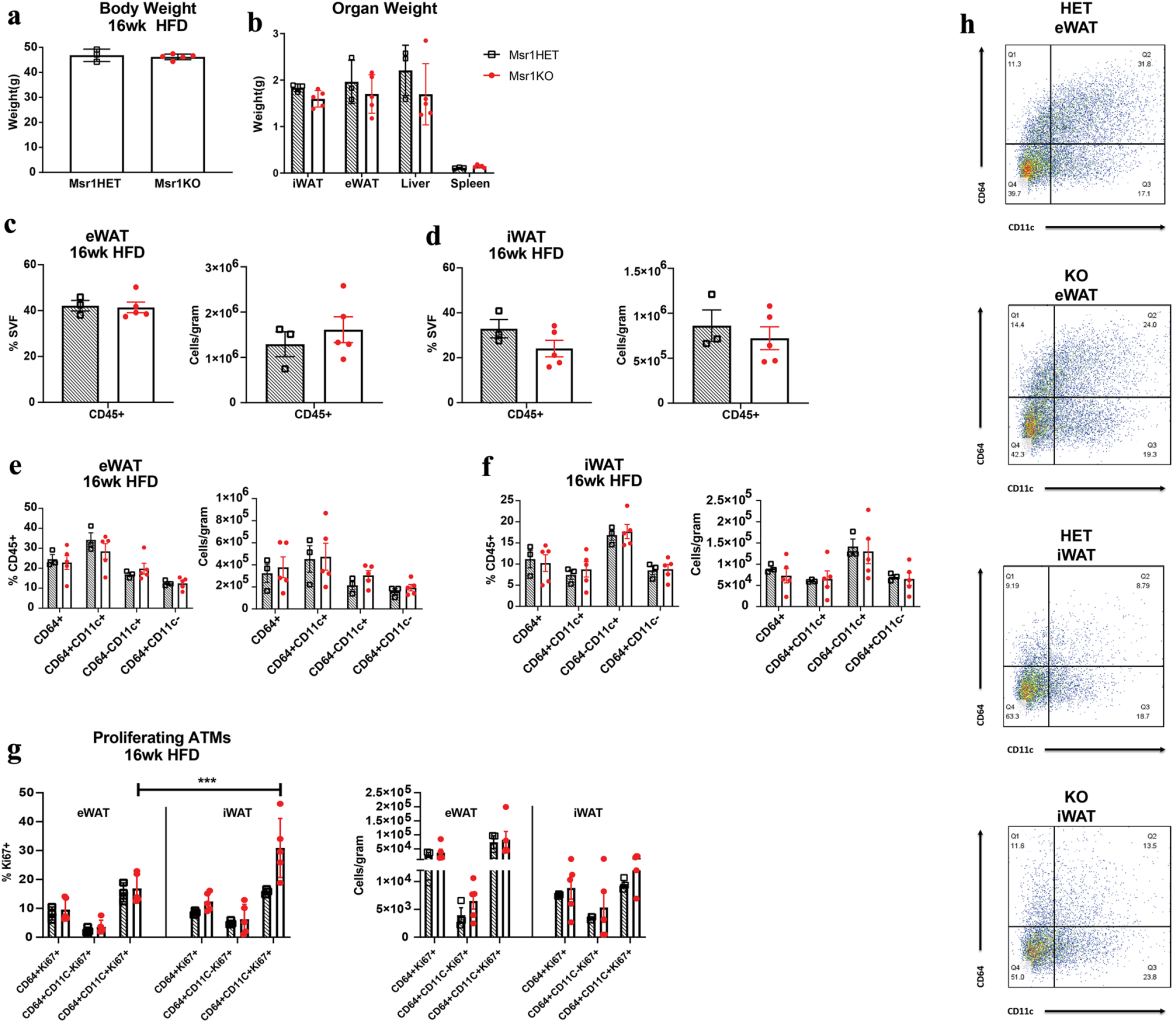
Msr1 is not required for ATM infiltration during obesity in obese male mice. (**a**) Whole body weight and (**b**) organ weight for 16-week male HFD-fed Msr1HET (black) and Msr1KO (red) mice. Flow cytometry analysis of CD45^+^ cells in the SVF (**c**, **d**); ATM subtypes (**e**–**f**); and proliferating ATMs (**g**) in eWAT and iWAT. (**h**) Representative flow cytometry graphs. n = 3–5/group. ***p = 0.0005. Statistical Analysis = 2-way ANOVA multiple comparisons.

We examined if *Msr1* was required for obesity-induced ATM infiltration and proliferation. After 16 weeks of HFD, the SVF was isolated from fat pads and flow cytometry performed to quantify ATM subtypes and assess ATM proliferation. Both genotypes had similar body weights and there were no significant differences between spleen, eWAT, and liver weights between genotypes (Fig. 4a, b). *Msr1*KO mice had a trend for smaller iWAT fat pads compared to *Msr1HET* (*p*-value = 0.078). Flow cytometry analysis of SVF revealed no differences in the quantity of CD45 cells between *Msr1*-deficient mice and littermate controls in either eWAT or iWAT (Fig. 4c, d). Further analysis of the CD45^+^ leukocytes in the SVF revealed that *Msr1KO* and *Msr1HET* mice had the same frequency of total CD64^+^ ATMs, CD64^+^CD11c^−^ resident ATMs, CD64^+^CD11c^+^ inflammatory ATMs, and CD64^−^CD11c^+^ ATCDs (Fig. 4e–f). We quantified proliferating ATMs in both genotypes by Ki67 staining. We analyzed CD64^+^Ki67^+^, CD64^+^CD11c^−^Ki67^+^, and CD64^+^CD11c^+^Ki67^+^ ATMs from eWAT and iWAT and no significant differences in proliferating ATMs were observed between genotypes (Fig. 4g). We observed that there was a significant higher proportion of proliferating CD64^+^CD11c^+^Ki67^+^ ATMs in iWAT compared to eWAT in *Msr1KO*.

**Figure 5:**
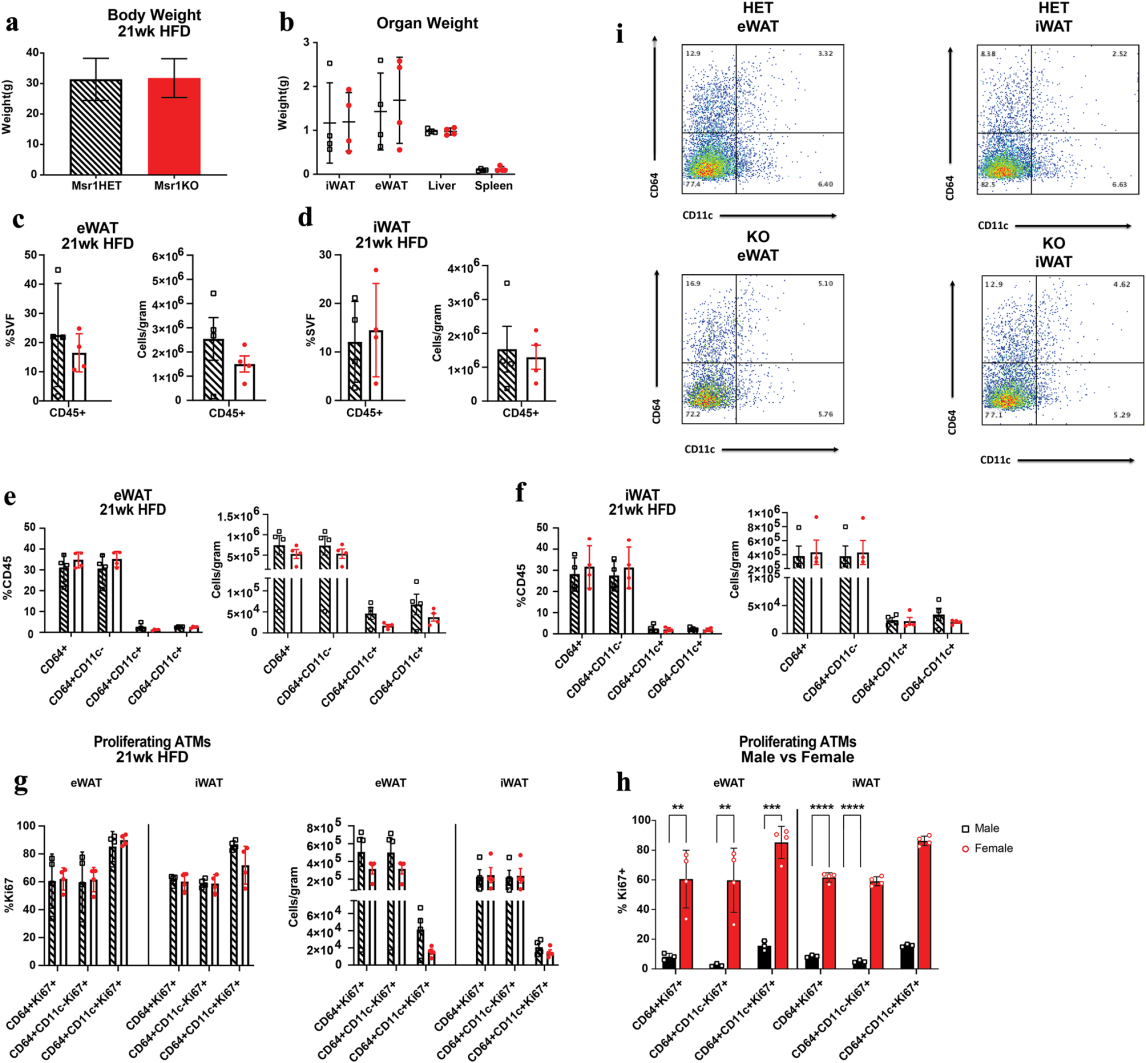
Msr1 is not required for ATM infiltration during obesity in obese female mice. (**a**) Whole body weight and (**b**) organ weight after 21 weeks of HFD, at the time of euthansia. Flow cytometry analysis showing the frequency of (**c**, **d**) CD45^+^ cells in SVF, (**e**–**f**) %CD45 cells, and (**g**) proliferating ATMs in eWAT and iWAT from female *Msrt1HET* and *Msr1KO* mice. (**h**) Quantitation of proliferating ATMs in HFD fed male and female *Msr1HET* mice. (**i**) Representative flow cytometry graphs for (**e**–**f**). n = 3–5 per group. Statistical Analysis = t-test.

Similar analyses in female mice revealed no difference in body weight or organ weight after 21 weeks of HFD. (Fig. 5a, b)*.* Quantitation of ATMs by flow cytometry after 21 weeks of HFD demonstrated no differences in total CD45^+^ leukocytes in eWAT or iWAT between genotypes (Fig. 5c, d). HFD fed female mice fail to induce significant CD11c^+^ ATMs (Fig. 5e–f) compared to male mice, but there were no differences between genotypes observed in total or subset ATM content. Similar levels of Ki67^+^ proliferating ATMs were observed between genotypes (Fig. 5g). However, when compared with male mice, HFD female mice had a greater frequency of proliferating total CD64^+^Ki67^+^ ATMs and subtypes in both iWAT and eWAT (Fig. 5h). Overall, these observations indicate that *Msr1* is not required for obesity-associated adipose tissue inflammation and ATM infiltration and demonstrates that ATM proliferation is more robust in females compared to males.

## Discussion

This study aimed to elucidate the role of *Msr1* in insulin resistance and adipose tissue inflammation during obesity. The premise of the study is based on our initial observations that that *MSR1* is increase in the VAT of obese, diabetic humans and correlates with BMI in GTEX in both VAT and SAT. Analysis of published single nuclear RNA sequencing datasets demonstrate that *MSR1* expression is highly restricted to ATMs and may be a biomarker for total ATM content in human adipose tissue. We were able to observe increased ATM proliferation in HFD fed female mice compared to males by flow cytometry analysis. We observed this in both subcutaneous and visceral adipose tissue depots and related to increased proliferation in all subtypes of ATMs. This result support previous observations of increased female ATM proliferation using Ki67 staining in adipose tissue sections with lipolysis^33^. Why female ATM proliferation is more prominent in males is unclear. It is possible that an increased proliferative capacity in female mice is related to suppression in obesity-induced adipose tissue inflammation compared to males^34^.

Because we did not observe changes in ND male mice and because female mice do not exhibit the same degree of obesity as male mice^33,35^, experiments in female mice were only carried out in HFD-fed mice. Our observation of a lack of Cd11c + inflammatory ATMs in female adipose tissue despite significant hypertrophy is consistent with prior studies showing a blunted recruitment of ATMs in female mice. While initial cohorts suggested protection from obesity and impaired ATM proliferation in male *Msr1KO* mice this was not replicated in littermate controls. We were unable to replicate prior studies suggesting that *Msr1*-deficient mice were more susceptible to inflammation with HFD induced obesity and instead initially observed that *Msr1*-deficient mice were protected from HFD-induced obesity and adipose tissue inflammation. The difference in weight gain observed between *Msr1KO* and WT non-littermate controls may be the primary cause for the protection from obesity-induced inflammation observed in *Msr1KO* mice. Protection from DIO is a common feature in many mouse models where macrophage genes are knocked out including other scavenger receptors such as CD36 and may be due to increased energy expenditure^36–38^. The differences we observed in obesity development in our non-littermate experiments could be attributed to differences in the microbiome as a result of the separate housing conditions.

To ensure a more rigorous experimental design, we compared *Msr1KO* mice with *Msr1HET* littermate controls. While there is no standard model for HFD feeding to induce obesity, C57BL6 mice exhibit significant weight gain and insulin resistance between 16 and 22 weeks of HFD^39^. Since we observed differences in body weight between the genotypes in the non-littermate studies, we extended the duration of HFD feeding to 16 weeks to ensure weight gain in the littermate studies With this experimental design we observed that both male and female *Msr1HET* and *Msr1KO* mice exhibited the same degree of HFD-induced weight gain, insulin resistance, and adipose tissue inflammation. Our findings suggest that *Msr1* does not play a significant role in controlling obesity-associated insulin resistance and inflammation.

One limitation of the studies may be the use of *Msr1*HET littermates as the control group as it is possible that *Msr1*HET mice with half the expression of WT mice. Because *Msr1*KO mice had significantly less *Msr1* expression in their adipose tissue compared to their *Msr1*HET littermates, we still feel this rigorously tests the requirement for *Msr1* in HFD induced obesity. However, it is possible that *Msr1*HET have a phenotype that differs from WT mice although no groups have reported abnormalities in *Msr1*HET mice. COVID-19 pandemic restrictions did not allow us to examine littermate *Msr1HET*, *Msr1KO*, and *Msr1* + */* + littermates. We also attempted to use commercially available antibodies to verify Msr1 expression but were unable to get these reagents to detect Msr1 by immunofluorescence or flow cytometry (data not shown).

Using littermate controls instead of mice from a parallel but distinct colony is recommended in metabolic studies to ensure the epigenetic and environmental backgrounds are comparable^28^. Having the appropriate control is necessary to determine the phenotype of genetically modified strains and can be a significant source of variation in metabolic studies. The *Msr1KO* mice were developed from 129SV embryonic stem cells injected into C57BL/6 J blastocysts and are backcrossed to C57BL/6JIco mice. Studies found that 129 X C57BL/6 mice display spontaneous autoimmunity, and in mutant mice backcrossed to this strain the target gene is likely influenced by surrounding 129SV genes^40,41^. Therefore, it is possible that the effects of the mixed background are significant and need to be controlled for more rigorously. However, we did conduct experiments to directly test if *Msr1HET* littermates had an altered phenotype compared to C57BL/6 WT mice however, the results we obtained in this study are all similar to our experience with HFD feeding in C57Bl/6 mice in our colony^25,26^.

Our inability to replicate findings with *Msr1KO* mice from other studies may be due to other factors that influence mouse metabolism. A recent large-scale study comparing genetically identical mice (C57BL/6 J) housed at four separate Mouse Metabolic Phenotyping Centers (MMPC) demonstrated substantial variation in the response to identical HFD feeding paradigms based on location^42^. Significant differences in site-specific metabolic rates were observed suggesting that location-specific differences in housing conditions, equipment, microbiome, or other factors may contribute to variation in responses to HFD induced obesity. Regression analysis identified body composition, activity, photoperiod, diet and acclimation conditions contributed the most to variance in energy expenditure. Single nuclear RNA sequencing from human adipose tissue demonstrates that *MSR1* is highly specific to ATMs. The association between *MSR1* expression and DM status may be proportional to ATM content which is increased in DM patients based on our recent results and other studies^43^. We attempted to validate MSR1 protein expression in mouse adipose tissue by immunofluorescence and flow cytometry but failed to obtain specific staining with commercially available MSR1 antibodies.

Overall, our experiments do not support a functional role for MSR1 in obesity-induced adipose tissue inflammation despite being a biomarker for ATM content. Our initial hypothesis of a role for MSR1 in adipose tissue inflammation was based on prior research supporting roles for MSR1 in the regulation of antigen presentation^44^ and in lipid uptake in macrophages in the setting of fatty liver disease^27^. Given differences between mice and humans in ATM function, future studies should evaluate potential functional roles for MSR1 activity in human ATM function.

## Supplementary Information

Supplementary Table 1.

## Data Availability

The single nuclear RNA sequencing datasets generated and/or analyzed during the current study are available in the Single Cell Portal at #SCP1903^31^. The data used for the GTEX described in this manuscript are available at dbGaP accession number phs000424.vN.pN on 11/12/2022.
