# Supplementary material for: MSR1 is not required for obesity-associated inflammation and insulin resistance in mice
Source: Sci Rep. 2023 Feb 14;13:2651. doi: 10.1038/s41598-023-29736-0 (PMC9927046; doi:10.1038/s41598-023-29736-0)
Supplement: Supplementary file 1 — Supplementary Table 1. [file 41598_2023_29736_MOESM1_ESM.pdf]

Gene	Forward Sequence	Reverse Sequence
Msr1	5' – GCATGGCAACTGACCAAAGA – 3'	5' – CCATGTTCCTGGACTGACGAA – 3'
Olr1	5' – CAAGATGAAGCCTGCGAATGA – 3'	5' – ACCTGGCGTAATTGTGTCCAC – 3'
Cd36	5' – AATTAGTAGAACCGGGCCAC – 3'	5' – CCAACTCCCAGGTACAATCA – 3'
Tnfα	5' – GATCTCAAAGACAACCAACATGTG – 3'	5' – CTCCAGCTGGAAGACTCCTCCCAG – 3'
IL1β	5' - AAATACCTGTGGCCTTGGGC – 3'	5' - CTTGGGATCCACACTCTCCAG – 3'
IL-10	5' - GCTCTTACTGACTGGCATGAG – 3'	5' - CGCAGCTCTAGGAGCATGTG – 3'

Supplementary Table 1. Primers for qPCR
